# Heavy Metals and Trace Elements in Human Breast Milk from Industrial/Mining and Agricultural Zones of Southeastern Spain

**DOI:** 10.3390/ijerph18179289

**Published:** 2021-09-02

**Authors:** Miguel Motas, Sandra Jiménez, José Oliva, Miguel Ángel Cámara, María Dolores Pérez-Cárceles

**Affiliations:** 1 Department of Toxicology, Regional Campus of International Excellence “Campus Mare Nostrum”, Faculty of Veterinary, Campus of Espinardo, University of Murcia, 30100 Murcia, Spain; sjrejon@hotmail.com; 2 Department of Legal and Forensic Medicine, Biomedical Research Institute (IMIB), Regional Campus of International Excellence “Campus Mare Nostrum”, Faculty of Medicine, University of Murcia, 30100 Murcia, Spain; mdperez@um.es; 3 Department of Agricultural Chemistry, Regional Campus of International Excellence “Campus Mare Nostrum”, Faculty of Chemistry, Campus of Espinardo, University of Murcia, 30100 Murcia, Spain; josoliva@um.es (J.O.); mcamara@um.es (M.Á.C.)

**Keywords:** human breast milk, inorganic compounds, heavy metals, trace elements, environmental pollution

## Abstract

Human breast milk is the most complete foodstuff for infants but can also be a potential source of exposure to toxic chemicals. The aim of this study was to assess the levels of metal pollution in the breast milk of women living in agricultural and industrial/mining areas of the Region of Murcia (Spain) that are well known for their cases of environmental pollution. Human milk samples were collected from 50 mothers and inorganic contaminants were analyzed using inductively coupled plasma mass spectrometry (ICP-MS). The mean or maximum concentrations of the different inorganic elements analyzed in breast milk, with the exception of manganese, exceeded the maximum limits established by the WHO and could constitute a high risk for pregnant mothers and their children. The breast milk of women living in the industrial/mining zone presented the highest levels of aluminum, zinc, arsenic, lead, mercury and nickel. On the contrary, the highest concentrations of manganese, chromium and iron were determined in the milk of women living in the agricultural zone. These results suggested and confirmed different profiles of environmental contamination of these areas.

## 1. Introduction

Breast milk is the most complete and natural food that children can consume during the early stages of their life, as it ensures their proper nutrition and development. An exclusive breastfeeding diet for up to 6 months reduces diseases such as diarrhea or pneumonia and even infant mortality [1].

In addition, breastfeeding contributes to the health and well-being of mothers, helps to space children, reduces the risk of ovarian cancer and breast cancer, increases family and national resources, is a secure way of feeding and is safe for the environment [1]. It even exerts a protective effect when there are high concentrations of toxic substances, as stated by many authors [2,3]. 

Only in very specific cases have measures of restriction of breast milk been published [4]. Most of the toxic substances passed from mother to child are transferred transplacentally and, to a lesser extent, via breast milk [5,6,7]. Therefore, the high levels of certain contaminants in milk reflect environmental contamination and not a problem unique to breastfed children. Children are especially vulnerable to different inorganic elements since their detoxification mechanisms are not fully developed and their organs are in formation [4]. Maternal exposure to heavy metals such as Pb or Hg is associated with children’s neurodevelopment delay. The presence of heavy metals in milk—including arsenic, lead, cadmium and mercury—induces changes in the structure of the immune system and also in its function by disturbing the homeostasis. These inorganic elements cause stimulation or suppression of the immunomodulatory components and can indirectly influence various body organs as well as the nervous, reproductive, respiratory and endocrine systems. This can lead to health problems in children such as allergies, disorders in the endocrine system and even neurodevelopment delay and disorder [8]. 

The trajectory of one’s life may be significantly altered depending on where they were born, which may result in exposure to toxicants such as lead, which could subsequently affect biological systems (neurological, hepatic or cardiovascular), ultimately altering income, education level and occupation, for example. The life course approach may be explained by lead exposure during one’s lifetime, since exposure to lead increases with age; the pathologies associated with this exposure also increase with age [9].

This exposure starts early as lead crosses the placental barrier, and the fact that it competes with calcium causes it to affect fetal and maternal bone function. Maternal bone is an endogenous source of lead, as a woman who has accumulated lead during her lifetime may have a significant store of lead in her bones. This accumulation suggests that women who have been exposed to lead in the past are at risk of exposing the developing fetus to lead via cord blood and, after birth, through breast milk [9]. Therefore, there is no safe level of exposure to lead or a threshold for adverse effects.

Age at onset, duration and changes in the level of exposure to risk factors such as inorganic elements may alter their effects on adult disease risk and impact on long-term disease trends [10].

We are surrounded by thousands of substances toxic to humans, and milk constitutes a valuable method of analyzing these, serving as a monitoring mechanism for the environmental pollution that surrounds us.

In the southeastern area of the Murcia Region (Spain), over the last century, a large number of industries were derived from mining that obtained lead, silver and zinc, releasing significant amounts of lead and arsenic oxides into the atmosphere, among others [11]. The Bay of Portman is one of the oldest known mining sites, mainly of lead, zinc and others such as pyrite. However, operations were mainly carried out from 1962 onwards out in the open pit, leading to large spills [12]. Studies carried out in the area found a large number of chemical elements with high bioavailability—mainly cadmium, followed by lead and arsenic, among others [13]. In addition, the Escombreras valley industrial complex is also located in that area, which leads to emission of waste with high levels of mercury and caused the waters near the valley to be considered one of the most polluted areas in Europe by the United Nations and by the European Environment Agency.

In order to compare the levels of the different chemical substances found in the milk of mothers in the industrial/mining area, we analyzed a second group of samples from women residing in an agricultural area of Murcia where other chemical substances from fertilizers and pesticides predominate. Due to the characteristics of the study area, inorganic elements (metals and other chemical elements) have special relevance.

Differences in the levels of inorganic contaminants in breast milk may depend on multiple factors, such as maternal factors (anthropometric and sociodemographic characteristics, maternal habits, maternal feeding, etc.), infant factors (age or time of breastfeeding; differentiation depending on whether it is colostrum, transitional milk or mature milk; prematurity, etc.) and others, such as the sampling time (in the morning or at night, at the beginning or at the end of the feeding), the sampling method (collection and transport), environmental factors (level of contamination in the area, exposure or duration), analytical method, contamination of samples, etc. [14,15,16].

The aim of this study was to quantify the levels of heavy metals and trace elements in samples of breast milk from women living in industrial/mining and agricultural areas, assess the relationship between metal pollutants in breast milk and lifestyle and maternal characteristics and evaluate the toxicological risk of exposure to environmental pollutants according to different variables, for both mother and infant.

## 2. Materials and Methods

### 2.1. Samples

Fifty individual breast milk samples were obtained from nursing mothers (primiparous and multiparous women with mixed or exclusive lactation, aged 32.98 ± 4.04 years old, who had resided in the represented area for at least the previous 5 years) at a primary care center. The women were residents in the Region of Murcia, south Spain: Cartagena, La Union and Portman were the mining and industry locations, and San Javier was one of the agricultural areas, among others. Thirty percent (*n* = 15) of the mothers lived in an agricultural area during the last five years, while 70% (*n* = 35) lived in an industrial–mining area. Sixty-two percent (*n* = 31) gave exclusive breastfeeding and 38% (*n* = 19) alternated breast milk with formula milk. The mean age of babies in the milk collection period was 7.5 ± 8.5 months (range: 1–32). The mean number of months of breastfeeding, including the months of breastfeeding in previous children, was 10.51 months.

All the mothers chosen for the study were not occupationally exposed to chemicals. From each of the participants, information was collected about the characteristics of the mother (housewife, place of residence, exclusive breastfeeding, age, weight, body mass index (BMI), size, number of children, years living in an industrial and mining area and total months of breastfeeding in previous children), of the child (age, birth and current weight), maternal habits (consumption of tobacco, glasses of water a day) and maternal nutrition (consumption of fruits, fish, meat and vegetables). The characteristics of the mothers, children and maternal nutrition according to the group (industrial–mining area vs. agricultural area) are shown in Table 1.

The criteria and approach for donor selection and human milk sampling were based on the “Guidelines for Developing a National Protocol” [17]. Milk samples (about 50 mL) were hand-expressed or breast-pumped into 100-milliliter pre-cleaned polypropylene pots, and appropriate precautions (washed with a 2% solution of HNO3 quality Suprapur and Milli-Q water) were taken to prevent contamination of the samples. All samples were immediately frozen and kept at −20 °C until analysis. 

### 2.2. Materials and Reagents

The following materials and reagents were used through the study: Suprapur-grade nitric acid and hydrogen peroxide (Merck^®^); Milli-Q Plus Millipore double-distilled and deionized water; disposable plastic Pasteur pipettes; adjustable Finnpipette micropipettes (Thermo Electron Corporation, Waltham, MA, US, NA) and disposable plastic tips; screw cap containers; disposable plastic tubes of 15 mL, with snap caps; beakers; 25-milliliter volumetric flasks; Microwave Milestone Ethos Sel. model with Teflon vessels; an ultrasound bath; Agilent 7500 Series ICP-MS equipment with a CETAC ASX-510 autosampler; and commercial stock solutions (Merck^®^) for each of the elements.

### 2.3. Analysis

The analytical procedure for the extraction of inorganic elements from human milk samples was similar to that described by Jerez et al. [18]. Briefly, breast milk samples were processed in a Microwave Milestone, Ethos Sel. model, using the “organic up 0.4” digestion program (Table 2). To prevent contamination of the samples, the Teflon vessels used for the digestion of the samples were kept in acid solution (2% solution of HNO3 quality Suprapur and Milli-Q water) for 24 h and subjected to an ultrasound bath with heat for approximately 2 h (70 °C). The thawed samples were subjected to a wet digestion using a mixture of HNO_3_ (65%), H_2_O_2_ (30%) and double-distilled and deionized water in a ratio 5:2:3, respectively. The resulting digestion was brought to a volume of 25 mL with double-distilled and deionized water, remaining refrigerated at 4 °C until analysis by ICP-MS.

The analysis of the inorganic compounds was performed on an Agilent 7500 Series ICP-MS system with a CETAC ASX-510 autosampler, a Peltier-cooled Scott-type nebulizer chamber, a MicroMist concentric nebulizer, nickel cones, 27.12 MHz radio frequency generator and a 1600 W Fassel-type quartz torch, argon mass flow control in plasma, auxiliary line, adjustment line and carrier gas, a hyperbolic quadrupole mass filter (3 MHz and 2-260 amu) and a simultaneous digital/analog detector with 9 orders of magnitude of linear dynamic range and a collision/reaction cell.

The following isotopes were selected for the analysis of the elements: 27Al, 52Cr, 55Mn, 56Fe, 60Ni, 63Cu, 66Zn, 75As, 78Se, 111Cd, 202Hg and 208Pb. The sensitivity was Li (7) ≥ 8 Mcps/ppm; Y (89) ≥ 20 Mcps/ppm; Tl (205) ≥ 12 Mcps/ppm; background (for 5 amu) ≤ 5 cps; oxides (in CeO+) ≤1, 0%; divalent cations (in Ce^2+^) ≤3.0% with detection limits of Be (9) ≤ 2 ppt, In (115) ≤ 1 ppt and Bi (209) ≤ 1 ppt.

The detection limits of the analytical method were as follows: 0.004 μg g^−1^ (Al), 0.0002 μg g^−1^ (Cr, As, Hg), 0.0004 μg g^−1^ (Mn, Ni), 0.002 μg g^−1^ (Fe), 0.0008 μg g^−1^ (Cu, Pb), 0.003 μg g^−1^ (Zn), 0.0007 μg g^−1^ (Se) and 0.0001 μg g^−1^ (Cd). Gallium and rhodium were used as internal standards.

Quality control of the procedure (reproducibility and reliability of the results) was carried out by introducing the samples in duplicate at random, with blanks at the beginning of each series of analyses and every 5 samples. The calibration standards were analyzed initially and periodically. Seven standards of each of the elements were used that were analyzed from commercial stock solutions (Merck^®^), which were stabilized with 20 μL of HNO3. The concentrations of the standards were 1, 5, 10, 25, 50, 100 and 200 ng/L. In cases where the upper limit of the calibration line was insufficient to detect levels higher than this in the test samples, standards of a higher concentration or dilution of the sample in question were prepared. The correlation index (R2) of the calibration lines was equal to or greater than 0.999.

### 2.4. Statistical Analysis

The results of the maternal/child characteristics, maternal habits and nutrition and inorganic element concentrations in breast milk were collected in a database (Microsoft Access 11.0; Microsoft corporation, Seattle, WA, USA), and statistical analysis was performed using the SPSS 24.0 software (SPSS Inc., Chicago, IL, USA). 

Normality and homogeneity of variance were tested prior to application of the analysis. The Mann-Whitney U-test or the Kruskal-Wallis test was performed to analyze group differences on continuous or ordinal data. Differences between groups on categorical variables were analyzed using Pearson’s χ^2^ or Fisher’s exact probability test. Results were expressed as mean ± standard deviation, median and range or as a percentage. Spearman’s rho correlation was used to measure the association between two quantitative variables. *p*-values below 0.05 were considered significant.

## 3. Results and Discussion

The descriptive statistics of the heavy metal and trace element levels found in the breast milk samples are shown in Table 3. Likewise, the percentages that exceed the maximum tolerable limit (MTL) of the different inorganic compounds in breast milk as established by the WHO are detailed with respect to the total of the samples [19], except for aluminum. However, in the case of aluminum, the FAO/WHO established the tolerable daily intake (TDI) as a toxicity control parameter.

An infant’s daily consumption of breast milk can vary depending on the child’s age and its solid food intake. In this study, the calculation of the daily and weekly intake for infants was made considering an average daily rate of milk consumption of 800 mL/day per infant during the first six months of life and a child’s body weight of 6 kg as reported in the WHO Infant/Child Growth chart (2009) [20]. The daily and weekly intakes were calculated using the following formulas:

$$\begin{array}{l} \mathrm{Daily}\;\mathrm{intake}\;\left(\frac{\mu g}{\mathrm{kg}\;\mathrm{body}\;\mathrm{weigth}}\;\mathrm{day}\right)=\frac{\mathrm{milk}\;\mathrm{intake}\;\left(\frac{L}{\mathrm{day}}\right)x\;\mathrm{contaminant}\;\left(\frac{\mu g}{L}\right)}{\mathrm{body}\;\mathrm{weigth}\;\left(\mathrm{kg}\right)}\\ \mathrm{Weekly}\;\mathrm{intake}\;\left(\frac{\mu g}{\mathrm{kg}\;\mathrm{body}\;\mathrm{weigth}\;}\;\mathrm{week}\right)=\frac{\mathrm{milk}\;\mathrm{intake}\;\left(\frac{L}{\mathrm{day}}\right)x\;\mathrm{contaminant}\;\left(\frac{\mu g}{L}\right)}{\mathrm{body}\;\mathrm{weigth}\;\left(\mathrm{kg}\right)}\;x\;7\;\left(\mathrm{days}\right) \end{array}$$

Overall, the mean concentrations of the heavy metals and trace elements investigated were higher than those recommended (Table 3). 

Lead was detected in 15 samples at higher levels than those reported in studies carried out in Chile [21], Greece [22], Slovakia [23], Sweden [24], Spain [25], Portugal [26], Italy [27] and Mexico [28]. The mother with the highest level of lead in her milk corresponds to a 28-year-old multiparous woman who has resided in the industrial/mining area for more than 5 years, with a university degree, who smokes or has smoked in the past and with a nursing child of 3 months. The weekly intake would be 83.62 μg/kg/week, a value higher than the reference of 25 μg/kg/week established by the EFSA [29]. 

Cadmium was detected in 3 of the 50 samples with a mean concentration similar to those found in breast milk samples from women in Poland [30], lower than those in Lebanon [31] and higher than those in Sweden [24], Greece [22], Italy [27] and Brazil [32]. The three samples exceeded the maximum recommended limits. The mothers with cadmium in their milk were women aged between 32 and 36 years, with normal weight (BMI around 22), two of them resident in an agricultural area and the other in an industrial/mining area, working outside of the home, with a smoking habit and with one-month-old children.

Mercury was detected at a mean concentration higher than that referenced in different studies carried out in Austria [33,34], China [35], Germany [36,37], Sweden [38], Slovakia [23,39], Turkey [40], Cyprus [41] and Brazil [32]. Twenty-seven samples exceeded the maximum tolerable limit. The mother with the highest level of mercury in her milk was a 38-year-old woman with normal weight, with two children, a housewife who had worked in greenhouses in the past, residing in the industrial/mining area, consuming fish weekly and with a 6-month-old infant.

Copper was detected at a mean concentration higher than the concentrations in other work published in Poland [30,42], Sweden [24], Portugal [26], Italy [27] and Saudi Arabia [43] but lower than those found in Nigeria [44], Greece [22] and Turkey [45]. Eighteen samples exceeded the maximum tolerable level of copper. The mothers with the highest levels of copper in their milk were women over 35 years of age with normal weight, multiparous, mostly residents in the agricultural area, with daily fruit consumption and weekly potato intake and vitamin intake only in pregnancy.

Zinc was detected in 36 samples, of which 11 exceeded the permitted intake limit. The mean zinc levels were higher than those found in other studies in Santiago, Chile [21]; Arica, Chile [21]; Sweden [24]; Portugal [26] and Italy [27]. The mothers with the highest levels of zinc in milk were women aged between 26 and 35 years old and of normal weight, all residents in the industrial/mining area, with prolonged breastfeeding (more than 20 months), except a mother with only one month of breastfeeding with vitamin consumption only in pregnancy.

Arsenic was detected in six samples. The mean values found are higher than most of those found in the reviewed literature [32,41,46,47]. All samples with arsenic exceeded the maximum tolerance limit. The mother with the highest level of arsenic in milk was a 36-year-old woman with normal weight, primiparous, who works outside the home, resides in the industrial/mining zone, with weekly fish consumption, a smoker and with a nursing son of 1 month.

Iron was detected in 33 samples with a mean concentration higher than in most publications [22,24,32,48,49,50]. Nine samples exceeded the maximum allowed level (350–720 μg/L). The mother with the highest level of iron in milk was a 37-year-old woman with normal weight, with two children, a housewife, a resident of the agricultural area, without intake of iron supplements and with a 24-month-old nursing son.

Thirty-nine mothers had detectable levels of nickel in their milk. Compared with the levels found in other studies, these results are higher than those of most authors [24,32,51,52]. The mother with the highest level of nickel in milk was a 36-year-old multiparous woman residing in the industrial/mining zone, who works outside the home, taking vitamin supplements during pregnancy and breastfeeding, with a baby of 3 months and a total of 3 months of breastfeeding, including the time for previous children.

Chromium was detected in 49 samples at a mean concentration higher than those found in other works [24,32,46,53,54,55,56,57]. Forty-six samples presented levels above the allowed limit. The mother with the highest level of chromium in milk was a 37-year-old multiparous woman, living in the agricultural area, working outside the home, consuming vitamin complexes during pregnancy and a smoker with a 24-month-old infant.

Selenium was detected in 37 samples at a mean concentration higher than those in other studies [24,26,32,58,59,60,61,62,63]. Thirty samples showed levels above the allowed limit. The mother with the highest level of selenium in milk was a 36-year-old multiparous woman residing in the industrial/mining area, working outside the home, consuming dairy products daily and meat several times a week, with intake of vitamin supplements during pregnancy and lactation and with a child of 3 months and a total of months of breastfeeding including the time for previous children of 3 months. This same mother had had the highest levels of aluminum and nickel in her milk.

Manganese was detected in eight samples at a mean concentration higher than that in different published studies [24,32,47,53,64,65]. Six samples exceeded the maximum limit allowed. The mother with the highest level of manganese in milk was a 38-year-old woman with normal weight, with two children, a housewife who in the past had worked in greenhouses, residing in the industrial/mining zone, with consumption of meat and fish weekly and of dairy daily, with exclusive breastfeeding and with a 6-month-old suckling child. This mother also had the highest level of mercury in her milk.

### 3.1. Relationship between Levels of Inorganic Pollutants and Characteristics of Mothers 

The relationships between the heavy metals and trace elements studied and the most significant characteristics of mothers are shown in Table 4.

The highest levels of aluminum, nickel, selenium, arsenic, lead, zinc and mercury in breast milk were found in women residing in the industrial and mining zone, which may be due to the fact that the study area is strongly affected by these anthropogenic activities, which make it the zone containing the most severely metal-contaminated sediments in the Mediterranean area [66,67]. However, the highest levels of chromium, iron, copper and manganese were found in women who resided in the agricultural zone, although these differences were not statistically significant. Regarding age, a positive and significant correlation (Spearman’s correlation coefficient (*rs* = 0.425; *p* = 0.002) was observed with the level of aluminum in breast milk, similar to that reported by Mandić et al. [68], probably by bioaccumulation. No statistically significant correlations were observed between the items and housewife status, weight, height, BMI, number of children and total months of breastfeeding in previous children.

Residence time in the industrial/mining area positively correlated with the concentration of nickel in breast milk (*rs* = 0.493; *p* < 0.001). Exclusive breastfeeding positively correlated with manganese levels—i.e., its concentration increases if breastfeeding is exclusive (*p* = 0.003). The concentrations of arsenic and cadmium were significantly lower in the milk of mothers who exclusively breastfed compared to mothers who fed their children with mixed lactation (*p* = 0.008 and *p* < 0.001, respectively). In exclusive breastfeeding, the feeding of the child is not complemented with other formula milks, with the milk production being greater, and therefore, there is maternal elimination of heavy metals [69]. García-Esquinas et al. [70] found the same result for cadmium.

### 3.2. Maternal Diet and Habits

The relationships between the heavy metals and trace elements studied and the most significant diet and habits of mothers are shown in Table 4. Mothers with a higher daily water intake had higher levels of lead and aluminum in their milk than those who consumed less water. Industrial spills and old plumbed pipes can cause lead to be elevated in drinking water, which could explain this fact. An Egyptian study found the same results [71]. On the other hand, the intake of aluminum through drinking water is generally low. However, we found that the aluminum levels in the breast milk of women who consumed the most water per day were significantly higher than those in mothers who consumed the least amount of water daily; as water is sometimes treated with aluminum salts during the drinking water production process, this could explain the increase in this metal in relation to the amount consumed. 

Women who consumed fruit daily had higher levels of copper in their milk than women who consumed it weekly (*p* = 0.021). Copper is present in pesticides, and residues can be found in fruits, which may explain the values found [22].

The level of cadmium and arsenic in breast milk was significantly higher in vegetarian women compared to those who were not. For both arsenic and cadmium, plants are one of their sources of exposure, which would explain this result. Leotsinidis et al. [22] found higher levels of cadmium in the milk of mothers with high vegetable consumption. The Mann-Whitney U-test showed that lead and manganese levels are significantly higher in non-vegetarian women.

Despite the fact that fish intake is considered the main source of mercury of non-labor origin, we did not find significant differences in the levels of mercury in the milk of women with high consumption of fish in general, shellfish or tuna (*p* = 0.539). These results coincide with those published by García-Esquinas et al. [70] and Gundacker et al. [34], probably due to the homogeneity in the consumption of the sample or due to the low passage into breast milk of the organic mercury present in the fish [6]. The concentrations in breast milk of the inorganic elements were not significantly different according to the consumption of meat or fish. However, despite the differences not being statistically significant, an increase in lead and mercury concentrations was observed with higher frequency of fish intake (levels of lead, mean ± SD (μg/L), according to fish consumption: never, 2.61 ± 8.41; 1–3 times a month, 6.74 ± 17.74; weekly, 7.75 ± 25.67, *p* = 0.570; levels of mercury, mean ± SD (μg/L), according fish consumption: never, 3.91 ± 5.87; 1–3 times a month, 3.75 ± 4.67; weekly, 10.97 ± 23.42, *p* = 0.539).

It was observed that mothers with current or past smoking habits had higher levels of cadmium in their milk than those who did not, coinciding with the results of many other authors [23,30,70,72,73,74,75]. Tobacco contains high concentrations of cadmium (0.1 to 0.2 µg per cigarette) that is easily absorbed through the respiratory tract [76]. Other works did not find such differences [22,75]. Arsenic was also positively correlated with cigarette smoking by mothers. Tobacco contains derivatives of arsenic as an irritant [77]; for this reason, it is reasonable to think that this habit in the mother would increase the levels of the toxin in breast milk. Chao et al. [75] found no such differences.

With regard to smoking, significant differences were observed in cadmium concentration (*p* = 0.014) between women who smoked or who had smoked at some point in their lives and non-smokers, with the cadmium concentration being higher in smokers.

### 3.3. Children’s Characteristics

Regarding the sex of the baby, no significant differences were observed in the concentrations of inorganic elements between boys and girls. A negative and significant correlation was observed between the concentrations of zinc and cadmium (*rs* = −0.392, *p* = 0.005 and *rs* = −0.353, *p* = 0.012, respectively) and the baby’s age and birth weight (Table 5). 

Cadmium was negatively correlated with the age and current weight of the child (*rs* = −0.294; *p* = 0.038), coinciding with the results of other authors [22,75,78]. The older the child is, the greater the milk production is and the greater the transfer of cadmium and zinc from the mother to the child is, thus depleting the maternal deposits. On the other hand, the milk of the first months contains higher amounts of proteins, to which cadmium and zinc are strongly bound; as lactation progresses and the milk is more mature, these decrease along with the inorganic elements [26,75]. These results coincide with those of other studies showing that the zinc concentration is higher in the first six months of breastfeeding [30]. Örün et al. [74] found this same negative correlation between cadmium levels and weight, although only in girls, while Ursinyova and Masanova [23] did not find such a relationship. Copper was negatively and significantly correlated with the current weight of the baby (*rs* = −0.335; *p* = 0.017). Arsenic concentration showed a significant negative correlation with birth weight in our study (rs = −0.392; *p* = 0.005). Arsenic, like cadmium, crosses the transplacental barrier, causing lower weight in the newborn [79].

The main limitation of the study is the limited sample size. However, most studies have a similar sample size [8], probably due to the difficulty of collecting these samples, their nature and ethical implications. As this is the first study of these characteristics carried out in an area of special contamination by inorganic elements, we believe that the data reported may be of interest to the scientific community.

## 4. Conclusions

This study quantifies the levels of heavy metals and trace elements in samples of breast milk from women in industrial/mining and agricultural areas. The relationship between metal pollutants in breast milk and lifestyle and maternal characteristics was assessed, and the toxicological risk of exposure to environmental pollutants according to different variables was evaluated, for both mothers and infants. The mean–maximum concentrations of the different inorganic elements analyzed in breast milk exceeded the concentrations recommended by the WHO and other international organizations, which could constitute a high risk for pregnant mothers and their children. The breast milk of women living in the industrial/mining zone for more than 5 years presented the maximum levels of aluminum, zinc, arsenic, lead, mercury and nickel, and the number of years living there was positively correlated with the levels of nickel. On the contrary, the highest concentrations of manganese, chromium and iron were determined in the milk of women living in the agricultural zone. These results suggested and confirmed different profiles of environmental contamination of these areas.

The levels of cadmium and zinc in breast milk were lower in mothers with older children, which would confirm the theory of the passage of toxic substances from the mother to the child during lactation, thus reducing their presence in the mother. Cadmium and copper levels were higher in the milk of mothers of children with lower current weight; in turn, arsenic levels were higher in the milk of women with children of a lower birth weight, which would confirm that exposure to high concentrations of certain toxic substances is associated with low infant weight.

Diet significantly influences the levels of certain elements in breast milk—for example, the amount of water consumed by the mother was associated with higher concentrations of aluminum and lead; vegetarian diets were associated with higher levels of lead, arsenic, cadmium and manganese; and high consumption of fruit was associated with an increase in copper concentrations. Promoting and defending breastfeeding is the responsibility of society as a whole, and being able to maximize its benefits means minimizing the mother’s and newborn’s exposure to different pollutants. Thus, actions must be taken to reduce and control pollutants by the competent authorities, as well as limiting certain habits and foods that are not recommended due to their high content of toxic substances.

## Figures and Tables

**Table 1:** Characteristics of the mothers, children and maternal nutrition according to the group (industrial–mining area vs. agricultural area).

Mother Characteristics	Industrial/Mining Area	Agricultural Area	*p*
Age (years) (mean ± SD) *	32.57 ± 4.25	33.93 ± 3.43	0.297
Weight (kg) (mean ± SD) *	67.17 ± 12.43	58.96 ± 8.59	0.018
Size (cm) (mean ± SD) *	163.82 ± 6.08	162.33 ± 4.22	0.517
*N* of children (mean ± SD) *	1.91 ± 0.70	1.40 ± 0.50	0.013
Housewife (n, %) **	11 (31.4)	2 (13.3)	0.163
Exclusive breastfeeding (n, %) **	24 (68.6)	7 (46.7)	0.341
Years living in the area (mean ± SD) *	19.48 ± 12.83	15.26 ± 12.48	0.265
Total months of breastfeeding in previous children (mean ± SD) *	13.22 ± 15.83	7.33 ± 12.65	0.083
**Child characteristics**			
Age (months) (mean ± SD) *	6.60 ± 7.65	9.73 ± 10.20	0.343
Birth weight (g) (mean ± SD) *	3312.25 ± 473.70	3105.33 ± 433.68	0.178
Current weight (g) (mean ± SD) *	7202.15 ± 3039.05	6217.13 ± 3786.23	0.452
**Maternal habits**			
Smoker or ex-smoker (n, %) **	12 (34.3)	5 (33.3)	0.608
Glasses of water a day (n, %) ***			0.375
<4	4 (11.4)	0	
4–5	13 (37.1)	7 (46.7)	
≥6	18 (51.4)	8 (53.3)	
**Maternal nutrition**			
Fruit consumption (n, %) **			0.666
Weekly	2 (5.7)	1 (6.7)	
Daily	33 (94.3)	14 (93.3)	
Vegetable consumption (n, %) **			0.476
Weekly	3 (8.6)	2 (13.3)	
Daily	32 (91.4)	13 (86.7)	
Blue fish consumption (n, %) ***			0.083
Never	12 (34.3)	10 (66.7)	
1–3 times a month (n, %) ***	14 (40)	2 (13.3)	
Weekly	9 (25.7)	3 (20)	
White fish consumption (n, %) ***			0.560
Never	2 (5.7)	2 (13.3)	
1–3 times a month	6 (17.1)	1 (6.7)	
Weekly	27 (77.2)	12 (80)	
Meat consumption (n, %) ***			0.165
Never	2 (5.7)	3 (20)	
1–3 times a month	6 (17.1)	5 (33.3)	
Weekly	22 (62.9)	5 (33.3)	
Daily	5 (14.3)	2 (13.3)	

SD: standard deviation, * Mann-Whitney *U* test, ** Pearson chi-square, *** Fisher test.

**Table 2:** Characteristics of the Microwave Milestone (Ethos Sel. Model) and “organic up 0.4” program.

Microwave Milestone: Ethos Sel. Model.			
Characteristics	“Organic Up 0.4” Program		
Closed System	Time (min)	T (°C)	Power (W)
6-sample capacity	0	20	-
High-pressure rotor	5	85	700
HPR 1000/6M	3	145	500
Automatic temperature sensor	30	210	1.000

T—temperature.

**Table 3:** Descriptive analysis of the levels of inorganic elements (μg/L) and percentage that exceeds the MTL established by the WHO.

Inorganic Elements	Min	Max	Mean	SD	P25	Median	P75	Detection Percentage	Percentage that Exceeds the Maximum Tolerable Limit (MTL μg/L) Established by the WHO	Tolerable Daily Intake (TDI) (μg/kg/day)
**Al**	0	882.4	34.3	133.0	0	0	0	18%	18% *	1000
**Zn**	0	7511.1	1402.6	1742.7	0	901.9	1851.3	72%	30.5% (2000)	300
**As**	0	15.3	0.9	2.71	0	0	0	12%	12% (0.6)	0.3
**Cd**	0	7.8	0.4	1.6	0	0	0	6%	6% (1)	0.4
**Pb**	0	89.2	5.2	16.7	0	0	1.9	30%	12% (5)	3.6
**Hg**	0	83.6	5.6	12.4	0	2.4	6.5	58%	54% (1.7)	0.7
**Cr**	0	454.7	16.1	63.6	3.3	5.4	10.1	99%	92% (1.5)	0.9
**Mn**	0	450.0	10.7	63.6	0	0	0	16%	12% (4)	140
**Fe**	0	7205.8	679.1	1387.3	0	195.7	518.5	66%	18% (720)	114
**Ni**	0	212.5	25.3	33.8	2.5	17.8	34.6	78%	54% (16)	12
**Cu**	0.93	1217.6	368.5	301.0	160.2	262.8	486.3	100%	36% (310)	71.4
**Se**	0	273.0	44.5	49.5	0	35.1	64.6	74%	60% (24)	30

* Tolerable daily intake (TDI) established by the FAO/WHO; SD: standard deviation, P25: 25th percentile, P75: 75th percentile.

**Table 4:** Levels of inorganic elements in breast milk (μg/L) according to characteristics, diet and habits of mothers.

	Place of Residence			Exclusive Breastfeeding			Glasses of Water A Day				Vegetarian			Fruit Consumption			Smoker		
Inorganic Elements	Agricultural	Mining	*	Yes	No	*	<4	4–5	≥6	**	yes	no	*	Weekly	Daily	**	Yes	No	*
	Mean (SD)	Mean (SD)	*p*	Mean (SD)	Mean (SD)	*p*	Mean (SD)	Mean (SD)	Mean (SD)	*p*	Mean (SD)	Mean (SD)	*p*	Mean (SD)	Mean (SD)	*p*	Mean (SD)	Mean (SD)	*p*
**Al**	37.6 (84.1)	32.7 (153.5)	0.418	36.5 (138.5)	9.6 (19.2)	0.790	0 (0)	6.2 (28.1)	61.1 (180.3)	0.049	9.6 (19.2)	36.5 (138.5)	0.790	10.43 (18.1)	35.9 (137.1)	0.604	71.1 (220.4)	15.4 (41.6)	0.915
**Zn**	1096.6 (1583)	1560.1 (1823)	0.268	1462.6 (1795)	712.2 (778.5)	0.575	2090.1 (3636)	1282.0 (1202)	1389.4 (1783)	0.837	755.1 (636.4)	1458.9 (1800)	0.745	744.73 (653.2)	1445 (1785)	0.515	1163 (1163)	1525.8 (1982)	0.836
**As**	1.4 (2.6)	0.60 (2.8)	0.082	0.44 (1.4)	5.7 (7.3)	0.008	0 (0)	0.7 (1.9)	1.0 (3.3)	0.724	8.6 (4.6)	0.2 (0.93)	0.000	0 (0)	0.92 (2.8)	0.442	2.0 (4.25)	0.3 (1.1)	0.054
**Cd**	0.7 (2.2)	0.2 (1.3)	0.223	0.1 (0.7)	3.9 (4.4)	<0.001	0 (0)	0.23 (1.0)	0.5 (2.0)	0.797	5.0 (3.6)	0.0 (0.0)	<0.001	0 (0)	0.42 (1.7)	0.624	1.2 (2.7)	0.0 (0.0)	0.014
**Pb**	7.2 (18.2)	4.1 (16.1)	0.114	5.4 (17.4)	2.8 (3.2)	0.270	0 (0)	0.24 (0.6)	9.7 (22.4)	0.014	3.5 (2.4)	5.3 (17.5)	0.005	1.28 (2.2)	5.42 (17.2)	0.655	10.4 (26.0)	2.5 (8.4)	0.063
**Hg**	2.3 (3.2)	7.2 (14.9)	0.170	5.2 (12.5)	10.3 (12.0)	0.528	7.0 (8.9)	7.1 (18.3)	4.0 (5.7)	0.884	9.5 (9.5)	5.2 (12.7)	0.158	5.26 (4.9)	5.57 (12.8)	0.941	4.9 (6.4)	5.9 (14.7)	0.807
**Cr**	33.5 (108.8)	7.1 (4.7)	0.690	17.1 (66.2)	4.7 (1.5)	0.474	7.0 (2.8)	7.2 (4.6)	24.3 (88.0)	0.797	4.4 (2.1)	17.1 (66.2)	0.317	6.05 (4.9)	16.73 (65.5)	0.511	32.3 (108.9)	7.8 (6.6)	0.493
**Mn**	29.1 (108.7)	1.2 (4.1)	0.261	11.2 (66.4)	4.6 (6.1)	0.003	0 (0)	2.4 (5.9)	18.7 (88.1)	0.633	5.0 (7.0)	11.2 (66.3)	0.003	0 (0)	11.37 (65.6)	0.854	27.9 (108.8)	1.8 (6.1)	0.086
**Fe**	1081.7 (2031)	471.7 (868.4)	0.386	661.5 (1413)	881.6 (1194)	0.229	789.1 (1256)	471.1 (911.6)	822.0 (1701)	0.715	595.5 (842)	686.3 (1431)	0.584	105.03 (145.7)	715.71 (1424)	0.416	668.3 (1743)	684.6 (1195)	0.458
**Ni**	27.2 (26.3)	24.3 (37.5)	0.440	26.5 (34.9)	10.6 (8.1)	0.281	17.4 (23.79)	22.0 (15.8)	28.9 (44.1)	0.811	10.2 (2.2)	26.6 (35.0)	0.281	6.59 (8.8)	26.45 (34.5)	0.169	30.9 (51.4)	22.4 (20.1)	0.719
**Cu**	404.6 (365.8)	349.9 (266.0)	0.814	377.6 (304.2)	264.3 (276.0)	0.211	526.6 (448.2)	308.0 (166.3)	390.7 (353.5)	0.853	184.7 (62.1)	384.5 (308.4)	0.198	116.29 (42.2)	384.63 (303.4)	0.021	334.5 (304.1)	386.1 (302.6)	0.467
**Se**	33.5 (27.2)	50.2 (57.4)	0.489	43.7 (48.3)	53.7 (70.5)	0.914	90.62 (66.63)	41.38 (36.36)	39.7 (53.83)	0.203	22.3 (7.7)	46.4 (51.2)	0.367	53.82 (58.3)	43.90 (49.6)	0.695	49.2 (64.1)	42.1 (41.0)	0.893

SD: standard deviation. * Mann-Whitney *U* test. ** Kruskal–Wallis test.

**Table 5:** Correlations of levels of inorganic elements in breast milk vs. child’s age, birth weight and current weight.

Inorganic Elements	Age		Birth Weight	Current Weight
**Al**	r_s_	0.051	−0.214	−0.062
	*p*	0.726	0.136	0.669
**Zn**	r_s_	−0.392 **	0.151	−0.227
	*p*	0.005	0.294	0.112
**As**	r_s_	−0.269	−0.392 **	−0.250
	*p*	0.059	0.005	0.080
**Cd**	r_s_	−0.353 *	−0.249	−0.294 *
	*p*	0.012	0.081	0.038
**Pb**	r_s_	−0.072	−0.125	−0.160
	*p*	0.618	0.387	0.267
**Hg**	r_s_	−0.077	0.045	0.019
	*p*	0.596	0.759	0.897
**Cr**	r_s_	0.067	0.044	−0.083
	*p*	0.644	0.760	0.564
**Mn**	r_s_	−0.182	−0.118	−0.269
	*p*	0.206	0.415	0.059
**Fe**	r_s_	−0.045	−0.007	−0.056
	*p*	0.754	0.959	0.697
**Ni**	r_s_	0.166	0.010	−0.014
	*p*	0.249	0.948	0.924
**Cu**	r_s_	−0.205	0.140	−0.335 *
	*p*	0.152	0.331	0.017
**Se**	r_s_	−0.017	−0.017	−0.004
	*p*	0.909	0.909	0.977

r_s_: Spearman’s correlation coefficient. * *p* < 0.05; ** *p* < 0.01.

## Data Availability

The data presented in this study are available on request from the corresponding author.
